# Extracorporeal hyperoxygenation therapy (EHT) for CO poisoning: in vitro and in vivo feasibility of a full-scale batch system

**DOI:** 10.1038/s41598-024-84878-z

**Published:** 2025-02-03

**Authors:** Niklas B. Steuer, Hannah Lüken, Peter C. Schlanstein, Matthias F. Menne, Christiane Hoffmann, Cavan Lübke, Thomas Schmitz-Rode, Sebastian Victor Jansen, Ulrich Steinseifer, Rüdger Kopp

**Affiliations:** 1 Department of Cardiovascular Engineering, Institute of Applied Medical Engineering, Helmholtz Institute, Medical Faculty, RWTH Aachen University, Forckenbeckstraße 55, 52074 Aachen, Germany; 2 Department of Intensive Care Medicine, Medical Faculty, RWTH Aachen University, Pauwelsstraße 30, 52074 Aachen, Germany; 3 Institute of Applied Medical Engineering, Helmholtz Institute, Medical Faculty, RWTH Aachen University, Pauwelsstraße 20, 52074 Aachen, Germany

**Keywords:** Biomedical engineering, Preclinical research, Translational research

## Abstract

Carbon monoxide (CO) poisoning is one of the most common causes of injury and death from poisoning. The primary objective of therapy is to eliminate CO from the patient as quickly as possible to prevent acute and long-term effects. The ideal treatment is hyperbaric oxygen in a pressure chamber. However, pressure chambers are scarce, and therefore, the most common treatment is normobaric oxygen (NBO), which, however, has limited efficacy. Here, we present a full-scale batch system for extracorporeal hyperoxygenation therapy (EHT), which facilitates CO elimination extracorporeally by increasing the dissolved oxygen concentration in the blood. The EHT was characterized in vitro, resulting in a minimum carboxyhemoglobin half-life of 3.26 ± 0.11 min. In large animal trials the EHT reduced the median carboxyhemoglobin half-life by 42% (29.77 min EHT vs. 70.8 min control (NBO)). However, the EHT also induced oscillations in hemodynamic pressures due to changes in the animals’ circulatory volume during operation. After optimization, the EHT could be a promising option for treating CO poisoning.

**Supplementary Information:**

The online version contains supplementary material available at 10.1038/s41598-024-84878-z.

## Introduction

Carbon monoxide (CO) poisoning is one of the most common causes of injury and death due to poisoning worldwide^1^. The global incidence has remained stable over the past 25 years and is estimated at 137 cases per million, with a mortality rate of 4.6 cases per million^2^. In the US, accidental, non-fire related CO poisoning results in an annual societal burden of $ 1.3 billion^3^.

CO is produced during incomplete combustion. The colorless, odorless, and tasteless gas is undetectable by humans and, once inhaled, enters the bloodstream via the lungs. There it binds, competitively with oxygen (O_2_), to hemoglobin (Hb), creating carboxyhemoglobin (COHb)^4^. Unfortunately, the affinity of hemoglobin for CO is more than 200 times greater than that for O_2_^1^. For example, this means that a concentration of only 0.022% CO in the breathing air results in 30% of hemoglobin in the blood being occupied with CO (30% COHb)^5^. Consequently, oxygen delivery to organs and tissues is inhibited, resulting in hypoxia. In addition, the oxygen dissociation curve is shifted to the left, further inhibiting oxygen release from hemoglobin into the tissue^4^. CO also binds to cytochrome c oxidase, inhibiting mitochondrial respiration and causing additional apoptosis and neural necrosis^6^. Furthermore, CO causes platelet activation, an increase in reactive oxygen species and inflammation, contributing to neurological and cardiac injury^6^. Clinical symptoms of CO poisoning are nonspecific and range from headaches and dizziness to unconsciousness, coma, and death. Long-term symptoms occur in up to 50% of patients and include impairment of concentration, memory, learning, and speech, as well as depression, dementia, and psychosis^7,8^. Within 5 years, 10% of patients suffer a heart attack^9^, the risk of diabetes mellitus is increased^10^, and long-term mortality increases from 1.6 to 8.4%^11^.

The primary goal of therapy is to eliminate the CO from the organism to prevent acute and long-term effects^12^. CO poisoning is usually treated by administering normobaric oxygen (NBO) via face mask, which increases the elimination of carbon monoxide, decreasing the COHb half-life. Compared to breathing room air, which results in a COHb half-life of approx. 320 min, NBO reduces the COHb half-life to approx. 74 min^6^. A further decrease in the COHb half-life to approx. 20 – 40 min can be achieved by administering hyperbaric oxygen (HBO) inside a pressure chamber^6,13^. The underlying mechanism of CO removal is the increase in dissolved oxygen in the plasma, which shifts the equilibrium reaction towards more dissolved CO that can be eliminated. The basic concept was first described by Douglas et al.^14^. In a double-blind, randomized study by Weaver et al.^7^, HBO also reduced the incidence of cognitive sequelae from 33% to 18%. In contrast, Annane et al.^15^ found that in comatose patients, two HBO sessions worsened the outcome compared to one session. Buckley et al.^16^ could not establish an efficacy of HBO compared to NBO for cognitive sequelae in a Cochrane systematic review. A review by Roderique et al.^17^ even cautioned against HBO as potentially harmful due to a further increase in reactive oxygen species (ROS). In practice, the therapy is limited by the number of pressure chambers available, and the time required to prepare them before treatment. This can result in delays of several hours before treatment can begin^6,18,19^. In the US, for example, only 11.9% of 361 surveyed pressure chambers are quipped for dealing with emergencies^20^. In conclusion, new therapies and treatment options for patients with acute CO intoxication are needed^6^.

Currently, several new therapeutic options are under investigation. On the one hand, there are pharmacological approaches, including a combination of hydroxocobalamin and ascorbic acid in a reduced form^21^, a supramolecular complex (hemoCD)^22^, and molecules based on bioengineered neuroglobin^23,24^. They use scavenger molecules with high affinity for CO to eliminate CO from the organism. On the other hand, treatments based on extracorporeal membrane oxygenation (ECMO) are being developed. ECMO is traditionally used in severe cases of acute respiratory distress syndrome (ARDS) and chronic obstructive pulmonary disease (COPD) to support or take over the function of the lungs by exchanging respiratory gases extracorporeally^25^. Therefore, the use of ECMO to remove CO seems obvious. However, due to the high affinity of hemoglobin for CO, elimination must be enhanced to achieve sufficient efficacy. The approach of Fischbach et al.^26^, termed “photo-ECMO", centers on harnessing light irradiation to augment CO elimination. Fischbach et al. demonstrated the efficacy of their method by reducing the COHb half-life from 42.6 ± 1.5 min in the absence of light to 21.4 ± 1.4 min with the introduction of light. By increasing the blood flow rate and assembling six photo-ECMO devices in parallel, the COHb half-life was decreased even further to a minimum of 6.3 ± 1.2 min. Subsequently, Fischbach et al.^26^ amplified the partial oxygen pressure within their system to enhance CO elimination. This augmentation was achieved by elevating the sweep gas pressure to 1.33 atm (1.35 bar), yielding a COHb half-life of 5.2 ± 0.4 min when employing light irradiation in four parallel devices.

Our approach, extracorporeal hyperoxygenation therapy (EHT), is based on enhancing extracorporeal CO elimination by further increasing the dissolved oxygen concentration. Since treatment is applied only to the extracorporeally pooled blood, higher pressures can be achieved than in a pressure chamber, and the increase in ROS only occurs extracorporeally. In addition, the EHT system can be less expensive and more mobile than a pressure chamber, resulting in better availability and accessibility. In previous in vitro experiments, we investigated the approach in a small-scale batch system^27^. Here, we describe the newly developed full-scale model and the in vitro experiments as well as in vivo testing of feasibility in a large animal model. The high-pressure gas exchanger is the key component of the EHT system and is based on a bubble oxygenator^28^, in which bubbles are introduced into the blood to provide oxygenation and decarboxylation. For our high-pressure application, we developed a gas exchanger suitable for batch operation (see Fig. 6). A batch operation is favorable as it is possible to separate the gas exchanger from the extracorporeal circuit and therefore the patient during high-pressure detoxification. A standard batch operation with consecutive filling and emptying of the gas exchanger with blood would cause a circulatory volume shift for the patient each time, which is potentially harmful. We solved this problem by equipping the high-pressure gas exchanger with a blood loop containing two pumps and four valves (see Fig. 7). With different valve settings, the gas exchanger can be filled and emptied simultaneously while maintaining a constant blood flow in the extracorporeal circuit without circulatory volume shifts for the patient. Here, we describe the in vitro tests of a full-scale system and in vivo testing of feasibility in a large animal model.

## Results

### In vitro experiments

**Fig. 1:**
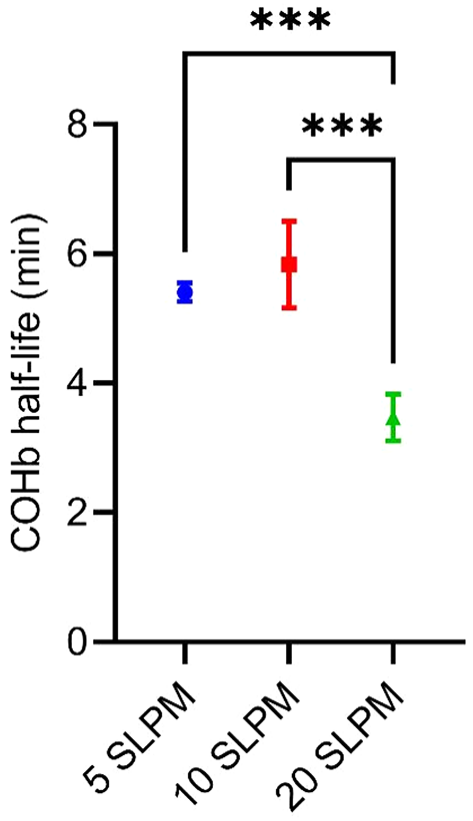
COHb half-life dependent on gas flow rate of oxygen through the gas exchanger. SLPM = standard liter per minute. The data is presented as mean ± standard deviation. (*) *p* ≤ 0.05, (**) *p* ≤ 0.01, (***) *p* ≤ 0.001.

The results of the in vitro experiments are shown in Fig. 1. The original data can be found as Supplementary Table S1 online. A gas flow rate of 20 standard liters per minute (SLPM) entailed the shortest COHb half-life (3.47 ± 0.36 min). Gas flow rates of 10 SLPM and 5 SLPM resulted in significantly longer COHb half-lives (5.84 ± 0.67 min and 5.4 ± 0.14 min, respectively), while the difference between both was not significant.

**Fig. 2:**
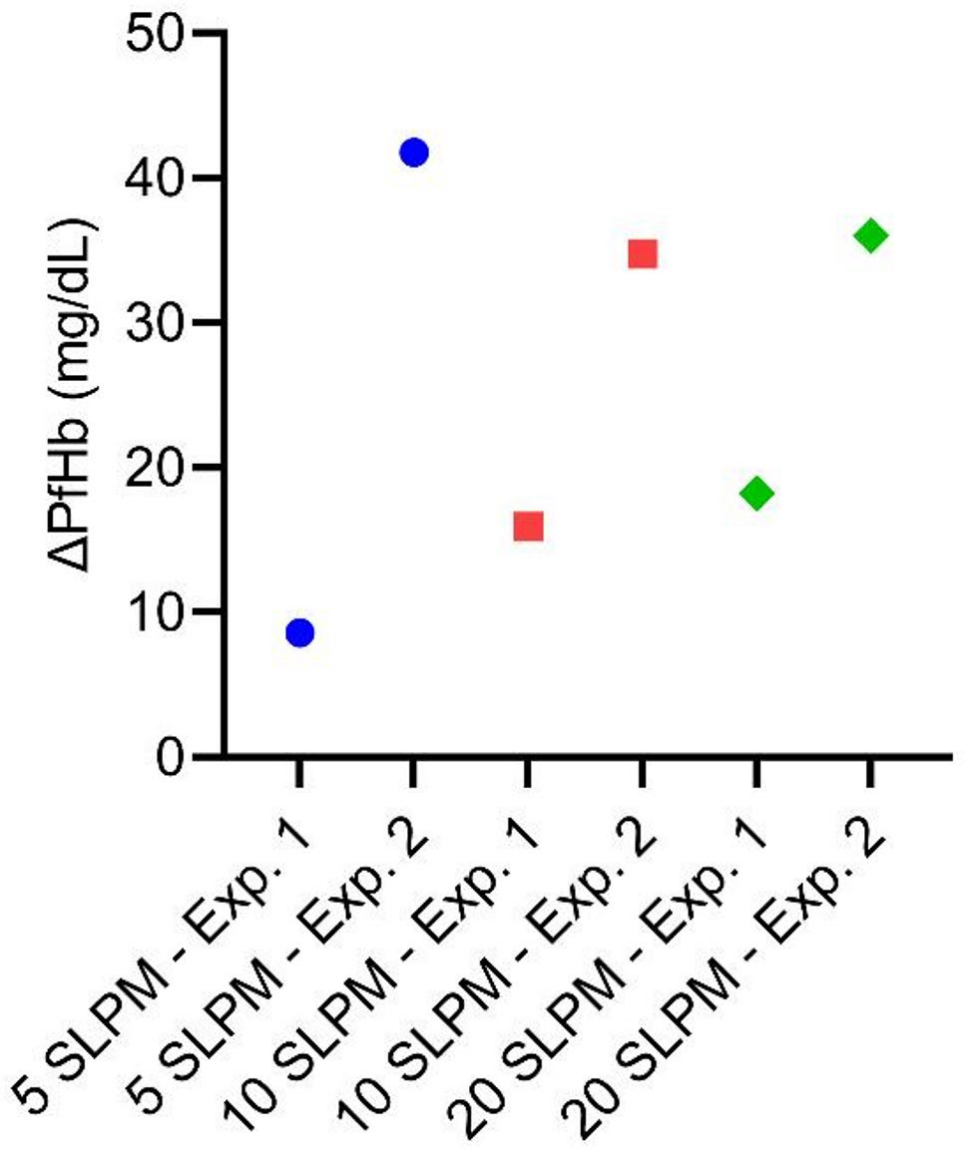
Difference of plasma free hemoglobin from pre- to posttreatment in a single batch dependent on gas flow rate depicted as data from single experiments.

To quantify the amount of hemolysis caused by the treatment, plasma free hemoglobin (PfHb) was measured. Figure 2 shows the difference in PfHb from pre- to posttreatment of a single batch with the EHT system. The original data can be found as Supplementary Table S2 online. Overall, PfHb was increased after treatment in a single batch, but there was no apparent dependence on gas flow. For example, for a gas flow of 5 SLPM, a difference in PfHb of 8.59 mg/dL was measured for the first experiment and 41.76 mg/dL for the second experiment. A similar variance was seen for the operating points with the other gas flows.

### In vivo experiments

**Fig. 3:**
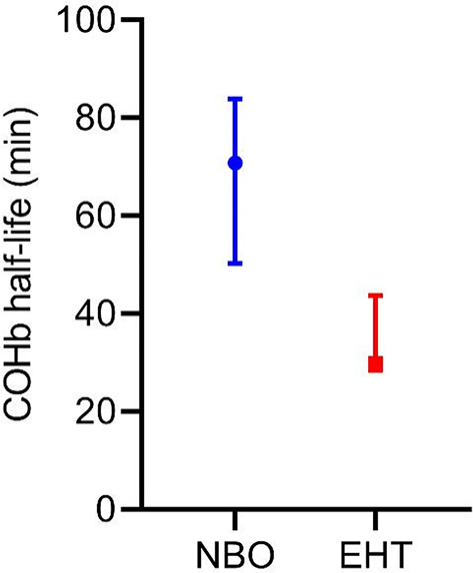
COHb half-life for treatment with NBO and EHT system in CO-poisoned pigs (*N* = 6; 3 – NBO, 3 - EHT). The data is presented as median and range.

**Fig. 4:**
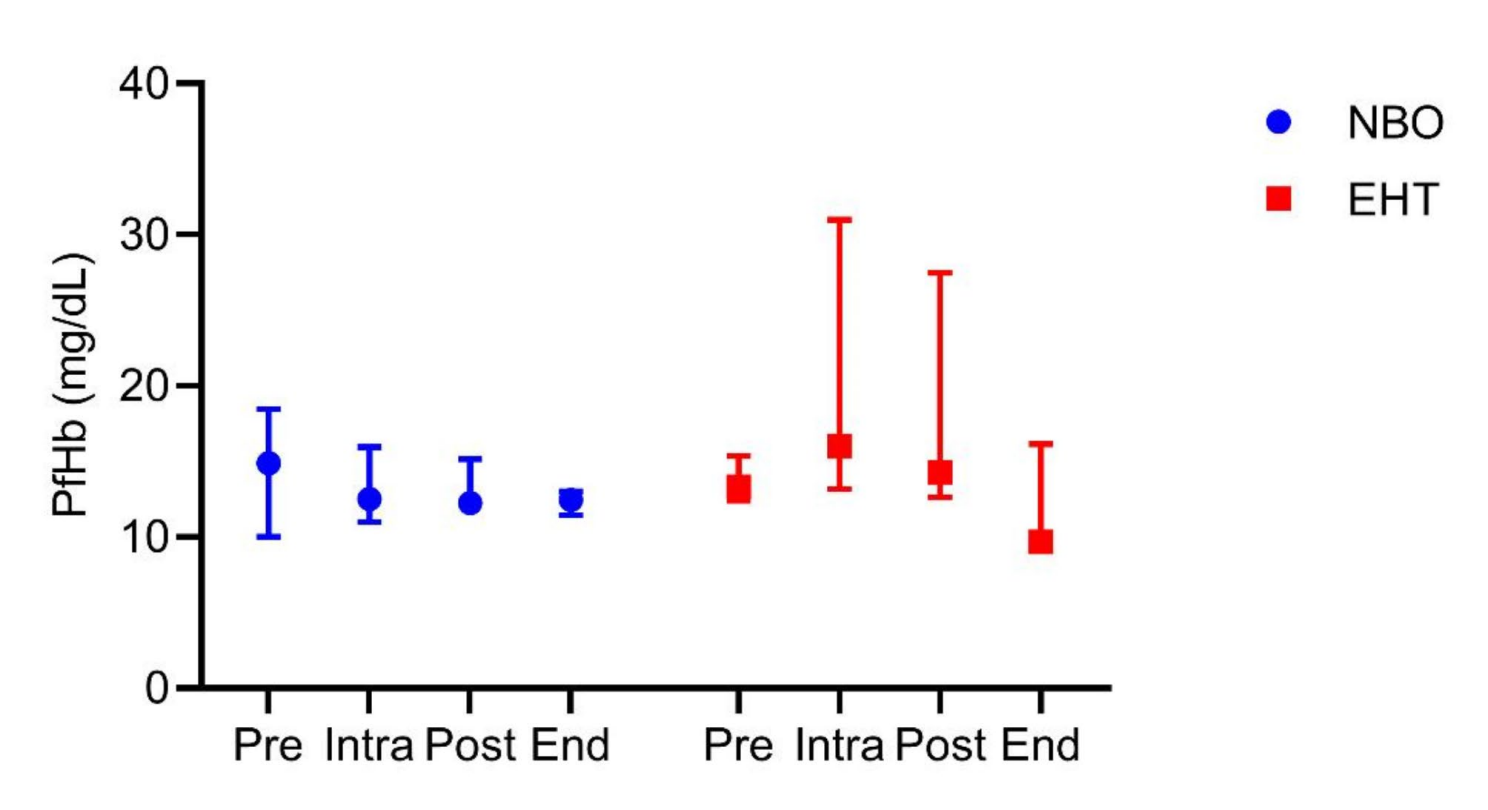
Plasma free hemoglobin (PfHb) at different times during the in vivo experiments: “Pre” at start of recovery phase, “Intra” at 60 min of recovery phase, “Post” directly after recovery phase, and “End” at termination of animal trial (360 min after start of recovery phase). (*N* = 6; 3 – NBO, 3 - EHT). The data is presented as median and range.

The results of the verification of the EHT in vivo showed a substantial decrease in the COHb half-life compared to NBO treatment alone (see Fig. 3). With the EHT, the COHb half-life was 29.77 (28.46, 43.72) min, whereas NBO treatment resulted in a COHb half-life of 70.8 (50.31, 83.91) min. Hence, the EHT system increased the CO elimination rate by a factor of 2.04. There was no distinct difference in median plasma free hemoglobin, although higher levels of plasma free hemoglobin were measured in one animal receiving EHT during and immediately after treatment. The results of the last measurement at the end of the trials showed comparable PfHb levels of the EHT group to those of the NBO group (see Fig. 4). The original data can be found as Supplementary Table S3 and S4 online.

**Fig. 5:**
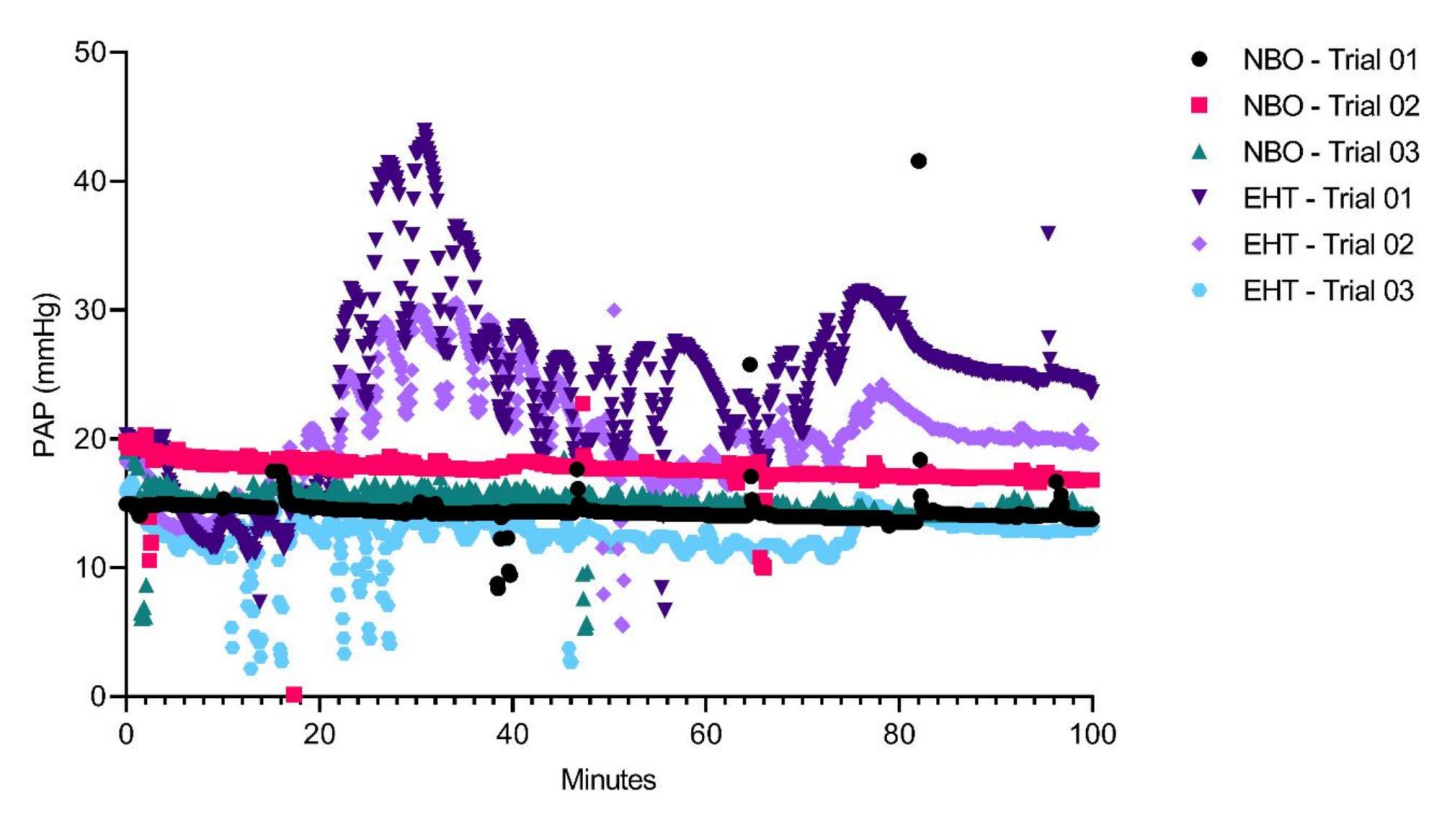
Mean pulmonary artery pressures (PAP) during the first 100 min of the recovery phase of the control (NBO) and the test (EHT) group. The EHT group was treated for 80 min with the EHT system.

As shown in Fig. 5, the EHT resulted in periodic oscillations in mean pulmonary arterial pressure (PAP) that correlated in frequency with the withdrawal and return of the blood. The same effects shown here for PAP were also visible in the arterial and central venous pressure values (see Supplementary Fig. S1 and S2 online). In the first two animals treated with the EHT system, this phenomenon resulted in significantly increased PAP levels, which normalized only slowly towards the end of the experiment. The amplitude of the pressure oscillations decreased with each treated animal. In the PAP values of the third animal, only very small oscillations could be detected, and the pressure normalized quickly after the end of EHT.

## Discussion

In this study, we present the prototype of a newly developed extracorporeal hyperoxygenation therapy system for the treatment of CO poisoning. The EHT system is designed to increase the dissolved oxygen concentration in the blood to accelerate the elimination of CO. This is accomplished in an extracorporeal device that treats one batch of blood at a time. In this batch, the dissolved oxygen concentration is greatly increased, facilitating the removal of CO. Subsequently, the treated blood is returned, and the process is repeated until the COHb value reaches physiological levels. The EHT system can be less expensive and more mobile than a pressure chamber, allowing for greater availability and accessibility. In previous in vitro experiments, we investigated the approach in a small-scale batch system^27^. Here, we describe the upscaled version of the system and a feasibility study. In the first part of the study, we characterized the CO elimination and blood damage of the EHT system in vitro. In the second part, we tested the EHT system in vivo in a large animal model imitating the clinical situation.

In general, the results of the in vitro experiments show that different gas flow rates influenced COHb half-life. The highest gas flow rate of 20 SLPM resulted in the shortest COHb half-life of 3.47 ± 0.36 min. This is most likely due to the continuously low concentration difference of CO between the blood and the gas phase. A higher gas flow rate removes the CO that diffuses into the gas phase more quickly so that a higher concentration difference between the blood and gas phases is maintained, compared to lower gas flow rates. The non-significant difference in COHb half-life for the gas flow rates of 5 SLPM and 10 SLPM is likely caused by a plateau or inflection point in the correlation. Since the CO elimination rate is indirectly proportional to the residence time, it can be assumed that the COHb half-life will decrease again at lower gas flows, indicating an inflection point. A corresponding trend can be seen in the results, although the difference is not significant.

In a previous study, in which we tested the CO elimination of a small-scale EHT system, the shortest COHb half-life was 21.32 min in a blood volume of 50 mL at a pressure of 7 bar and a gas flow rate of 0.3 SLPM. In comparison, in this study, the shortest COHb half-life was 3.26 ± 0.11 min in a blood volume of 500 mL at a pressure of 7 bar and a gas flow rate of 20 SLPM. The increase in CO elimination may be attributed to two effects. First, in the upscaled EHT system, the gas flow rate, as well as the ratio of gas flow rate and blood volume, was higher, which resulted in more gas bubbles in the blood and hence a larger surface area for the diffusion of CO out of the blood. Second, in the previous study with the small-scale system, the antifoam agent was applied directly to the surface of the blood, limiting the formation of bubbles. In the upscaled EHT system, we applied the antifoam agent to the filter foam, which was placed at the top of the inner cylinder. This created an empty space between the blood surface and the foam, in which bubbles consisting of a thin layer of blood filled with oxygen were generated by the gas flow. These bubbles also increased the surface area for the elimination of CO, which could account for the increased CO elimination of the upscaled EHT.

The most similar approach to our EHT system is the “photo-ECMO” described by Fischbach et al.^26^. By harnessing light irradiation and increasing the sweep gas pressure to 1.33 atm (1.35 bar) they enhanced the CO elimination in an extracorporeal hollow-fiber membrane oxygenator. Employing four devices in parallel, yielded a COHb half-life of 5.2 ± 0.4 min, compared to a half-life of 3.26 ± 0.11 min at 7 bar with our EHT system.

The hemolysis measurements indicate that the hemolysis of the EHT system varies widely. Our results show the system’s capability to operate with minimal generation of PfHb in certain instances, while concurrently demonstrating higher PfHb values in other experiments. These variations could also be attributed to variations in blood handling procedures, including the filling and emptying of the gas exchanger, as well as the treatment of individual samples. Notably, we observed no discernible correlation between hemolysis and gas flow rates, so that hemolysis considerations did not impact the determination of gas flow rates for the subsequent in vivo experiments.

The in vitro experiments have certain limitations that need to be considered. The range of gas flows investigated in the experiments was limited, and the observed results indicate the existence of potential inflection and plateau points. This implies that more favorable operating points for CO elimination may exist at gas flow rates not explored in this study. Additionally, the manual blood handling introduced variability, particularly evident in the hemolysis results. Furthermore, the limited number of hemolysis experiments conducted and the potential variations, e.g., in blood properties and experimental days, necessitate cautious interpretation. However, the EHT system was not specifically optimized for minimal hemolysis, and the intention of these experiments was to demonstrate the feasibility of operating at acceptable rates of hemolysis.

An animal study was conducted to further validate the feasibility of the EHT system, simulating the dynamics of clinical applications. When comparing the COHb half-life of the NBO treatment of the control group to values reported in the literature, our results show a COHb half-life of 70.8 (50.31, 83.91) min compared to 74 min reported in the literature^6^. Despite differences due to biological variation inherent to in vivo experiments, the established intoxication protocol provided a reproducible model for evaluating different treatments.

For the in vivo experiments, we selected a gas flow rate of 10 SLPM as our operating point. This was because a flow rate of 20 SLPM, which resulted in the lowest COHb half-life, would have reduced the detoxification time but made the manual part of the handling difficult during the experiments. This could have led to errors and failed experiments. Additionally, when considering the time it takes for the first batch of treated blood to be returned to the animal, a gas flow rate of 10 SLPM was more favorable than 5 SLPM due to the shorter detoxification time. In the in vivo experiments, the animals were mechanically ventilated, which continuously eliminates CO. Hence, the animal’s COHb continuously decreased while a batch of blood was being treated in the EHT system. Therefore, the following batch of blood drawn had a lower initial COHb level, resulting in a decrease in the amount of CO eliminated in the gas exchanger.

The in vivo performance of the EHT system resulted in a substantial reduction of the COHb half-life, indicating improved elimination compared to the control group. Importantly, CO elimination was twice as fast as with NBO alone, successfully translating the in vitro results to an in vivo setting. Fischbach et al.^26^ also achieved improved CO elimination rates with their photo-ECMO in in vivo trials, resulting in a 36% reduction in COHb half-life. However, comparison of our results with the in vivo results of Fischbach et al. is limited by differences in animal size, CO poisoning protocol (repeated poisoning cycles in a single pig vs. only one poisoning cycle per pig), and differences in baseline COHb levels. A comparison with pharmacological approaches is difficult, as the underlying mechanisms are different. In addition, the pharmacological approaches may be used in combination with extracorporeal systems to further enhance CO elimination.

Hemolysis is an important concern in the clinical application of extracorporeal circulation, as e.g. in ECMO it is associated with increased rates of endothelial failure, renal failure, thrombotic events, transfusions, non-hemorrhagic stroke, pulmonary and systemic hypertension, decreased organ perfusion and mortality^29,30^. In extracorporeal circulation, hemolysis occurs due to unphysiological shear stress that damages red blood cells, leading to the release of free hemoglobin into the blood plasma. PfHb scavenges nitric oxide (NO), which is essential for maintaining endothelial function and vasodilation; thus, its depletion can result in vasoconstriction, platelet activation, and an increased risk of thrombosis^29–31^. Furthermore, hemolysis entails the formation of reactive oxygen species that damage cell membranes, lipids, proteins, and DNA, inducing systemic inflammatory response syndrome (SIRS), and thus lead to tissue and organ damage^31^. In our experiments, hemolysis levels were maintained below the critical threshold of 50 mg/dL^32^ throughout the trials. In addition, hemolysis levels returned to normal after the EHT system was disconnected, indicating the system’s ability to operate without causing sustained blood damage. However, considering the risks associated with hemolysis, future development of the EHT system should aim to optimize hemocompatibility.

The oscillations in hemodynamic pressures, shown in Fig. 5 for pulmonary artery pressure, raise significant concerns about the clinical application of the EHT system in its current state. These oscillations were most pronounced in the first trial and showed a decreasing trend in subsequent tests. Therefore, we attribute the observed oscillations to the manual control of the pump speed during simultaneous filling and emptying of the reactor. This manual control resulted in a certain difference in filling and emptying speed of the inner and outer cylinder that may have caused a variation in volume in the animals’ circulation and thus the described oscillations. As the trials progressed, more experience was gained with the system, and the pumps were better synchronized, which explains the improvement between trials. Despite these improvements, small oscillations remained in the final trial, indicating the need for further optimization.

The nature of the EHT treatment includes dissolving large amounts of oxygen in the blood. As excess amounts of oxygen form bubbles during pressure release, there is a risk for introduction of these bubbles into the patient’s circulation, causing gas embolisms. In arterial circulation, gas embolisms may lead to ischemic events, neurological deficits, and, in severe cases, circulatory failure, cardiac arrest, and coma^33^. To reduce the risk of gas embolisms, hypobaric oxygenators have been developed for CPB and ECMO. These hypobaric oxygenators use sub-atmospheric pressures on the gas side to decrease gas concentration in the blood before reinfusion into the patient’s circulation^34^. Venous gas embolism is typically filtered by the lungs, but larger gas volumes can still cause cardiac arrhythmias, pulmonary hypertension, and right ventricular strain^33^. Due to the lower risk of venous gas embolisms, for dialysis machines Polaschegg and Levin^35^ do not classify a continuous infusion of air below 0.03 mL/(kg*min) and the infusion of a bolus of 0.1 mL/kg as dangerous. The EHT system also returns the treated blood into the venous circulation. Additionally, the debubbling protocol to prevent gas bubbles from entering circulation was developed. However, further studies are needed to verify its safety across different application scenarios.

An important limitation of this study relates to the animal model used, which was configured to mimic the standard therapeutic setup for humans, with a COHb half-life of approximately 70 min. While this approach provides a basic framework, it does not fully capture the range of scenarios encountered in real-life situations. The inherent variability in the severity and duration of CO exposure in clinical cases may affect the generalizability of the findings beyond the specific conditions simulated in this study. In addition, the study involved only 3 animals per group. While this size was considered acceptable for establishing the feasibility of the EHT system, a larger sample size would have contributed to improved statistical robustness and generalizability of the results. The inherent biological variability in animal models introduces an additional layer of complexity, as individual animals may have different responses to CO poisoning and treatment. In addition, we did not measure recirculation of previously treated blood back into the system during simultaneous filling and emptying. Increased recirculation would have negatively impacted the performance of the EHT system. Although the use of small cannulae and low flow rates potentially minimized the likelihood of significant recirculation, this should be considered when interpreting the study results.

In conclusion, the animal study represents an important step in validating the feasibility of the EHT system for CO elimination. The in vivo experiments demonstrated a substantial reduction in the COHb half-life with EHT compared to NBO treatment alone. The hemolysis results are acceptable, while there are still opportunities for improvement, for example regarding design, choice of materials, and production processes. Nevertheless, other aspects of hemocompatibility, such as thrombogenicity and inflammatory response should also be investigated in the future. However, the oscillations in hemodynamic pressures present a significant challenge for clinical translation. While improvements have been observed with increasing operator experience and adjustments, further optimization is essential to eliminate the oscillations. These optimizations could include servo-controlled pumps to match the blood flows or the redesign of EHT towards a continuous system. Following the optimizations, the EHT system, with greater availability and accessibility than HBO, could provide a promising option for managing CO poisoning. EHT might also be combined with veno-arterial extracorporeal membrane oxygenation (VA ECMO) in severe cases of CO poisoning where cardiac function is compromised, and VA ECMO is used to support heart and lung function^36^. The EHT system could be added to VA ECMO similar to how CRRT is combined with ECMO^37,38^, enhancing CO elimination while preserving hemodynamic stability. This combined approach could provide a safer, more feasible alternative to transporting ECMO-supported patients to hyperbaric oxygen chambers. Future studies could evaluate the practicality and efficacy of combining EHT with VA ECMO.

## Methods

### The system for extracorporeal hyperoxygenation therapy

**Fig. 6:**
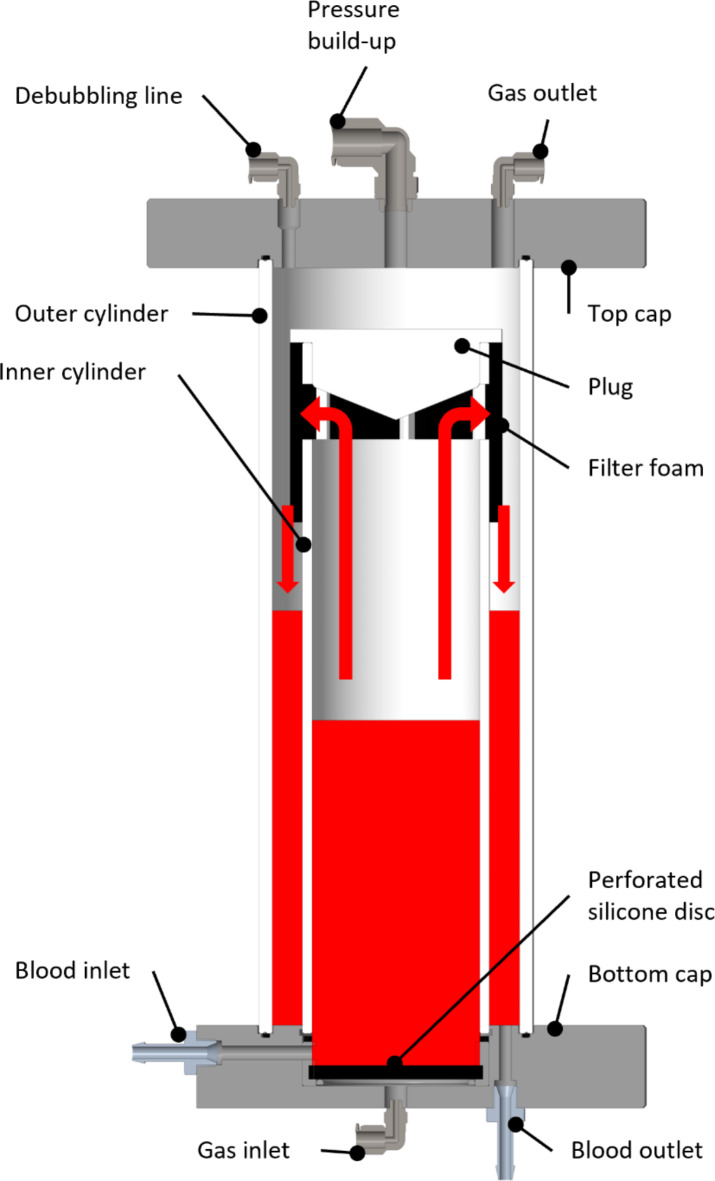
Detailed sectional view of the high-pressure gas exchanger. During operation, the blood is carried from the inner compartment along the red arrows into the outer compartment by oxygen bubbles that are introduced via the gas inlet.

**Fig. 7:**
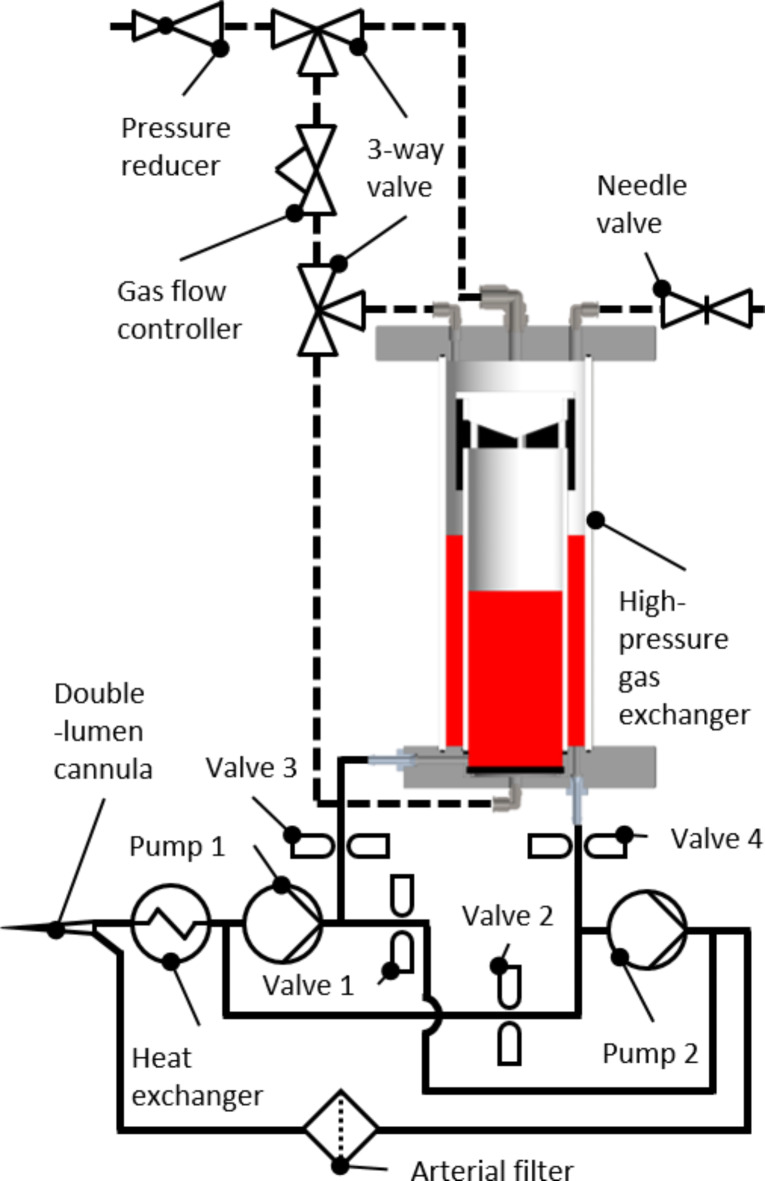
Schematic of the setup used in the in vivo experiments. The components were connected to a control system, allowing multiple batches to run automatically in sequence.

The high-pressure gas exchanger (see Fig. 6) had two coaxial compartments. The outer compartment was defined by the outer hollow cylinder with a length of 280 mm, an outer diameter of 100 mm and a wall thickness of 5.3 mm. The outer cylinder was fastened and sealed between two caps made from polyoxymethylene. Coaxial to the outer cylinder, a second inner cylinder (length 250 mm, outer diameter 75 mm, wall thickness 1.8 mm) was mounted on the lower cap, defining the inner compartment. The inner cylinder was closed at the top with a plug, and just below were six rectangular openings. A polyurethane filter foam (30 PPI, Filteron GmbH, Solingen, Germany) coated with Simethicone USP (Dow Corning^®^ Q7-2243 LVA, DuPont de Nemours, Inc., Wilmington, Delaware, USA) was wrapped around these openings on the outside of the cylinder. The bottom cap was equipped with a blood inlet and outlet, as well as a gas inlet. Above the gas inlet, a perforated silicone disc (OXYFLEX MT 300, Supratec Gesellschaft für Umwelt- und Verfahrenstechnik mbH, Simmern, Germany (cut to size)) was installed that dispersed the gas into the blood. The gas and blood inlets were connected to the inner compartment, and the blood outlet was connected to the outer compartment. The top cap was equipped with a gas oulet, a gas connector for pressure build-up and a gas connector for the debubbling line, which was a perforated Heidelberger line (B. Braun SE, Melsungen, Germany) that extended into the blood in the outer compartment. The gas section consisted of a pressure reducer connected to an oxygen bottle, two three-way valves, a gas flow controller, and a needle valve (see Fig. 7).

In the high-pressure gas exchanger, one batch at a time was treated according to the following procedure: 500 mL of blood was pumped through the blood inlet into the inner compartment, after which the inlet was clamped off via two-way valves and the pressure inside the gas exchanger was quickly increased by 6 bar by introducing oxygen into the system via the pressure build-up line. Subsequently, a gas flow (100% oxygen) was supplied via the gas inlet. The gas flow was dispersed by the silicone disc into the blood, creating bubbles in the blood in the inner compartment. The resulting dispersion rose inside of the inner cylinder until it was diverged by the plug through the openings. Once the dispersion contacted the filter foam outside of the openings, gas and blood were separated: the blood was collected in the outer compartment, while the gas escaped through the gas outlet. After the blood was carried from the inner to the outer compartment, the pressure inside the gas exchanger was released over 60 s. During the release of the pressure, 4 short (0.25 s) bursts of gas were passed through the debubbling line and dispersed into the blood to force nucleation and consequent release of excess dissolved oxygen. Once the gas exchanger had reached ambient pressure, a waiting period of 30 s succeeded to ensure the complete release of excess oxygen from the blood in the outer chamber. The debubbling procedure was optimized, until no bubbles were detectable with an ultrasonic bubble counter (BC100, GAMPT mbH, Merseburg, Germany).

To allow an automatic treatment of multiple batches successively without causing a shift in the patients’ circulatory volume each time, the high-pressure gas exchanger was expanded by a blood loop (see Fig. 7), controlled by a custom-made controller. The blood loop consisted of a 13 Fr double-lumen cannula inserted into a femoral vein (Achim Schulz-Lauterbach Vertrieb medizinischer Produkte GmbH, Iserlohn, Germany), a heat exchanger (ECMOtherm-II, Medtronic plc, Dublin, Ireland), two peristaltic blood pumps (15QQ, Boxer GmbH, Ottobeuren, Germany), an arterial filter (Baby Sherlock, EUROSETS S.r.l., Medolla, Italy), and four pinch valves. Two of the pinch valves (numbers 1 and 2) were regular pinch valves (ASCO S170XA01 × 2900VU, Emerson, Saint Louis, Missouri, USA), while the other two (numbers 3 and 4) were designed for high-pressure resistance (ASCO S206.05-Z130A-24VDC, Emerson, Saint Louis, Missouri, USA). The valves could be switched between two states: detoxification and simultaneous filling and emptying. During detoxification, valves 3 and 4 were closed to separate the high pressure in the gas exchanger from the blood loop. Valves 1 and 2 were open, and the blood low rate of both pumps was combined to provide the total blood flow rate of 300 mL/min, circulating the blood through the loop and the cannula. During simultaneous filling and emptying, valves 1 and 2 were closed to stop the blood circulation through the loop. Valves 3 and 4 were open, and the rotational speed of each pump was set individually to achieve the total blood flow rate each. Hence, the gas exchanger was filled and emptied simultaneously, while the total blood flow rate through the cannula remained constant. Once the inner compartment was filled and the outer compartment was empty, the simultaneous filling and emptying was stopped manually, and the system switched to detoxification.

### In vitro experiments

On the day of the experiment, pooled porcine blood was obtained from the slaughterhouse and fully heparinized. We used a CPB machine (HL-20, Maquet, Rastatt, Germany) to pump the blood through a circulation loop consisting of a cardiotomy reservoir and an oxygenator (hilite 7000, Xenios AG, Heilbronn, Germany). The blood was conditioned according to ISO 7199^39^, and afterwards, sweep gas containing 3% CO and 97% N_2_ (Linde AG, Pullach, Germany) was used to poison the blood to a target value of 40 − 45% COHb.

For the in vitro experiments, the high-pressure gas exchanger was operated without the blood loop. For every batch, we used freshly poisoned blood. After every batch, we collected the blood through the blood outlet and measured the COHb. During the experiment, the whole gas exchanger was placed in a water bath at 37°C to ensure a constant temperature. We varied the gas flow rate in the experiments to investigate the effect on detoxification. The variation in gas flow rates also resulted in different residence times because with lower gas flow rates, the blood rises more slowly in the inner cylinder and vice versa. The blood flow rates, resulting residence times, and number of replicates are shown in Table 1. All other parameters were kept constant. To measure hemolysis, we performed two experiments for each gas flow and analyzed the blood before and after. Each experiment was performed with fresh porcine blood.

**Table 1:** Gas flow rate during the experiments and resulting residence times.

Gas flow rate (SLPM)	Residence time (s)	Number of replicates (–)
5	240	6
10	120	11
20	60	6

### In vivo experiments

Following the in vitro experiments, we verified the feasibility of the EHT for CO poisoning in living organisms via in vivo experiments with single pigs. All experiments were performed in accordance with relevant guidelines and regulations, followed the principles of laboratory animal care and the authors complied with the ARRIVE guidelines. The experiments and the experimental protocol were approved by the governmental animal care committee (Landesamt für Natur, Umwelt und Verbraucherschutz Nordrhein-Westfalen, Recklinghausen, Germany). The experiments were performed on six female pigs of the German landrace with a weight of 76.9 ± 5.27 kg. Three pigs served as a reference to measure the elimination of CO under physiological conditions with normobaric high oxygen ventilation. The other three pigs in the test group received EHT for the treatment of CO poisoning. The animals arrived two weeks before the experiments for acclimatization and were examined by a veterinarian. 12 h before the start of the experiment, the animals were kept sober with free access to water. For premedication, they received 1 mL Atropin 1%, 4 mg/kg Azaperon i.m. and 10 mg/kg Ketamin i.m. Afterwards, intravenous access was placed in an ear vein, the animals were orally intubated, and anesthesia was maintained with 5 – 10 mg/kg propofol and 8 – 20 µg/kg fentanyl. Additionally, they received 1 ml/kg/h crystalloid fluids, and a urinary catheter was placed. The mechanical ventilation was set to a target paCO_2_ of 35 – 45 mmHg with a PEEP of 5 mbar, a respiratory rate of 10 – 18/min and a tidal volume of 6 – 10 ml/kg. An arterial catheter was placed into one femoral artery to continuously measure the arterial blood pressure and take blood samples, and a high-flow 13 Fr double lumen Shaldon catheter and a 4-lumen central vein catheter were placed into the femoral veins to connect the EHT system and to have central access to inject medication. Additionally, a pulmonary artery catheter was placed in the right internal jugular vein to continuously measure the pulmonary artery pressure and discontinuously measure the pulmonary capillary wedge pressure, and the cardiac output, as well as to take blood samples. Heparin was injected continuously with a target activated clotting time (ACT) of > 150 s; if necessary, boli were given to reach the target ACT.

**Fig. 8:**
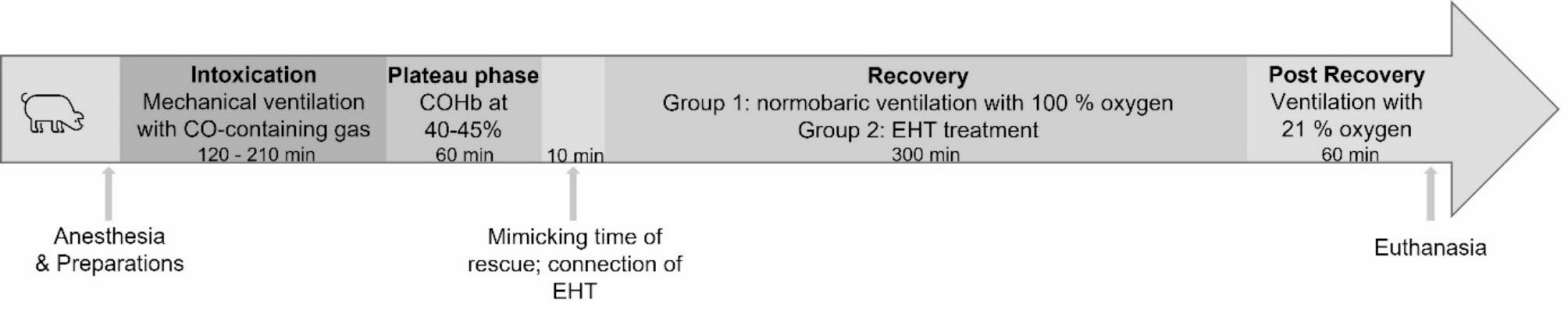
Experimental procedure for the in vivo experiments.

The experimental protocol included five phases: intoxication, plateau, rescue, recovery and post recovery (see Fig. 8). For intoxication, we connected a gas cylinder containing 940 ppm CO, 20% O_2_ and the rest nitrogen to the ventilator. FiO_2_ was set to 0.2 – 0.21. Blood samples for blood gas analysis were taken every 15 min to control the progress of intoxication, until the target COHb of 40 – 45% was reached. During the following plateau phase, the COHb was kept at 40 – 45% for 60 min by mixing air with the CO-containing gas and monitoring the COHb values via blood gas analysis every 10 min. Subsequently, the animals of both groups were ventilated with air (FiO_2_ 0.21) for 10 min, mimicking the rescue period of CO poisoned patients. During this phase, a bolus of 25,000 IU of heparin was administered, and the EHT system was connected to the animals of the test group via the Shaldon catheter. Before the connection to the cannula, the tubing and the outer compartment were primed with 0.9% saline solution to account for the additional extracorporeal volume of the EHT system. During the recovery phase, the control group received high oxygen ventilation (FiO_2_ 1.0) for 300 min, whereas in the test group, the elimination of CO was enhanced by the EHT. The target blood flow rate was 300 mL/min, resulting in a time of 100 s for simultaneous filling and emptying. The gas flow during detoxification was set to 10 SLPM. The different sequences and the respective durations for the in vivo experiments are summarized in Table 2. The EHT was continued for 75 min or until a target COHb of 5% was reached. Afterwards, the animals were ventilated with an FiO_2_ of 1.0 until the end of the recovery phase. Blood samples were taken every 15 min. Another period of 60 min was established as the post recovery phase to verify the lasting reduction of CO in the animals’ blood. During this period, the animals were ventilated with air. Blood samples for measuring COHb were taken every 15 min. Blood samples for measuring plasma free hemoglobin were taken every 60 min. Vital data was logged at a frequency of 0.2 Hz (temperature, heart rate, arterial pressure, central venous pressure, pulmonary artery pressure, central venous oxygen saturation) or recorded manually every 15 min (breathing rate, respiratory minute volume, inspiratory oxygen fraction, fractional end-tidal oxygen concentration, cardiac output, medication, pulmonary capillary wedge pressure).

**Table 2:** Different sequences and the respective durations for the in vivo experiments.

Sequence	Duration (s)
Filling and emptying	100
Detoxification	90
Pressure release	60
Waiting	30

### Analyses and statistics

The main parameter of interest was the fraction of COHb in percent. It was measured using a blood gas analyzer (ABL800 Flex, Radiometer Medical ApS, Brønshøj, Denmark). Hemolysis was determined by photometric measurement of the plasma free hemoglobin (Ultrospec 2100 Pro, Biochrom, Berlin, Germany). The analysis of hemolysis was performed according to DIN 58931^40^ by means of the cyanmethemoglobin method (Hemoglobin FS, DiaSys, Germany) according to the manufacturers’ instructions. For this, the plasma of each blood sample was separated from the cells by double centrifugation at 1,500×g for 15 min. The vital signs in the in vivo experiments were recorded at a frequency of 0.2 Hz with a custom-made data logger.

The COHb half-life was calculated using a nonlinear regression with a one phase exponential decay model and the assumption that the plateau equals 0% COHb. Subsequently, for the results of the in vitro experiments, we performed a one-way ANOVA followed by Tukey’s multiple comparison test. Normality was confirmed by Shapiro-Wilk test. Values are presented as mean ± standard deviation. For the results of the in vivo experiments, the variables are presented as median (range). All statistical analyses were performed with GraphPad Prism 9.

## Electronic supplementary material

Below is the link to the electronic supplementary material.

Supplementary Material 1

## Data Availability

The datasets generated and/or analyzed during the current study are available in this published article and its supplementary information files and from the corresponding author on reasonable request.
