# Supplementary material for: Extracorporeal hyperoxygenation therapy (EHT) for CO poisoning: in vitro and in vivo feasibility of a full-scale batch system
Source: Sci Rep. 2025 Feb 3;15:4066. doi: 10.1038/s41598-024-84878-z (PMC11790865; doi:10.1038/s41598-024-84878-z)
Supplement: Supplementary file 1 — Supplementary Material 1 [file 41598_2024_84878_MOESM1_ESM.docx]

Supplementary Information

for

Extracorporeal hyperoxygenation therapy (EHT) for CO poisoning: in vitro and in vivo feasibility of a full-scale batch system

Niklas B. Steuer^1^, Hannah Lüken^2^, Peter C. Schlanstein^1^, Matthias Menne^1^, Christiane Hoffmann^1^, Cavan Lübke^2^, Thomas Schmitz-Rode^3^, Sebastian Victor Jansen^1*^, Ulrich Steinseifer^1^, and Rüdger Kopp^2^

^1^ Department of Cardiovascular Engineering, Institute of Applied Medical Engineering, Helmholtz Institute, Medical Faculty, RWTH Aachen University, Forckenbeckstraße 55, 52074 Aachen, Germany

^2^ Department of Intensive Care Medicine, Medical Faculty, RWTH Aachen University, Pauwelsstraße 30, 52074 Aachen, Germany

^3^ Institute of Applied Medical Engineering, Helmholtz Institute, Medical Faculty, RWTH Aachen University, Pauwelsstraße 20, 52074 Aachen, Germany


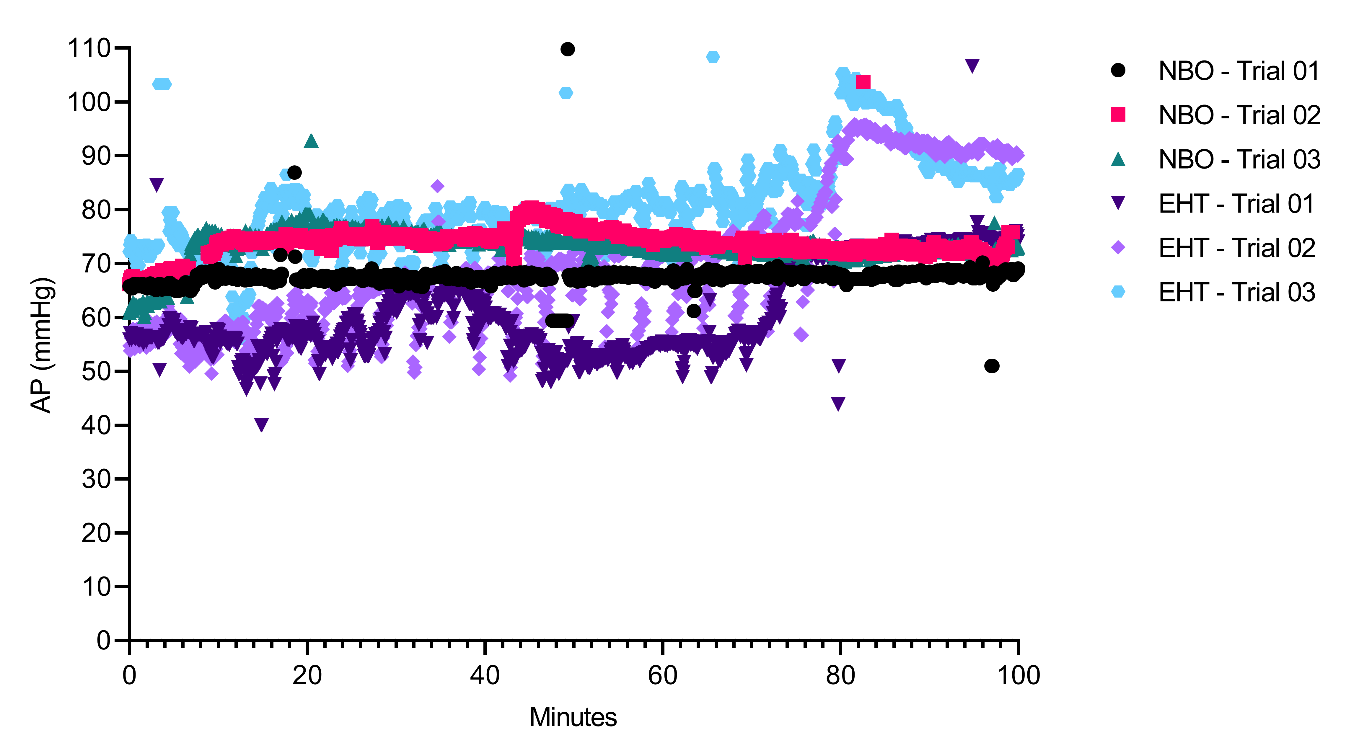


Supplementary Figure S1: Mean arterial pressures (AP) during the first 100 minutes of the recovery phase of the control (NBO) and the test (EHT) group. The EHT group was treated for 80 minutes with the EHT system.


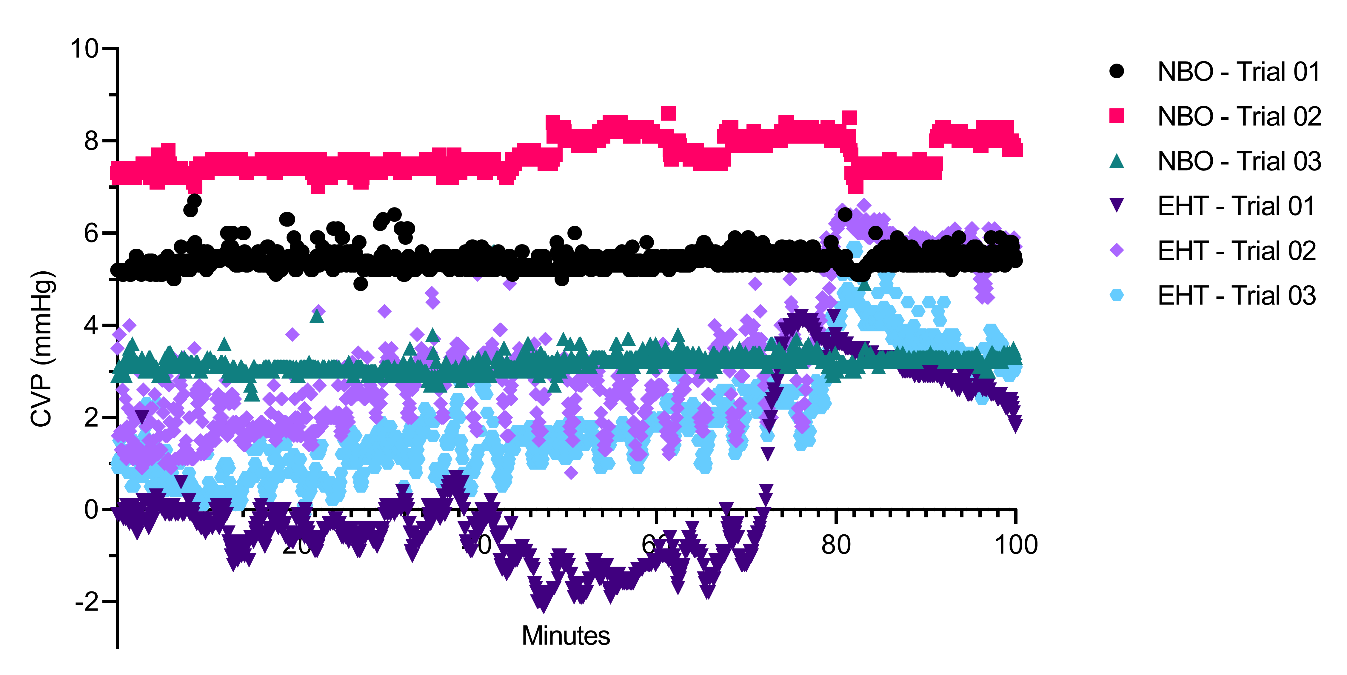


Supplementary Figure S2: Mean central venous pressures (CVP) during the first 100 minutes of the recovery phase of the control (NBO) and the test (EHT) group. The EHT group was treated for 80 minutes with the EHT system.

Supplementary Table S1: Carboxyhemoglobin (COHb) before and after the treatment in the EHT system in vitro with 3 different gas flow rates. Each gas flow rate resulted in the indicated treatment time of the batch.

| Gas flow rate |  |  | **COHb (%)** | | | | | | | | | | |
| --- | --- | --- | --- | --- | --- | --- | --- | --- | --- | --- | --- | --- | --- |
|  |  | **Time (min)** | **n=1** | **n=2** | **n=3** | **n=4** | **n=5** | **n=6** | **n=7** | **n=8** | **n=9** | **n=10** | **n=11** |
| **5 SLPM** | **pre** | **0** | 43,8 | 43,7 | 43,8 | 42,8 | 42,8 | 42,9 | - | - | - | - | - |
|  | **post** | **4** | 26,3 | 25,6 | 26 | 26,1 | 25,7 | 25,8 | - | - | - | - | - |
| **10 SLPM** | **pre** | **0** | 42,3 | 42,3 | 42,3 | 40,9 | 40,9 | 43,7 | 43,7 | 43,8 | 42,9 | 42,9 | 42,8 |
|  | **post** | **2** | 34 | 33,7 | 32,8 | 32 | 31,2 | 35,9 | 35,3 | 34,2 | 34,4 | 32,8 | 32,3 |
| **20 SLPM** | **pre** | **0** | 43,8 | 43,8 | 43,8 | 41,6 | 41,6 | 41,6 | - | - | - | - | - |
|  | **post** | **1** | 34,8 | 34,9 | 35,9 | 34,7 | 34,6 | 34,5 | - | - | - | - | - |

Supplementary Table S2: Plasma free hemoglobin (PfHb) before (Pre) and after (Post) the treatment in the EHT system in vitro with 3 different gas flow rates.

|  | **PfHb (mg/dL)** | | | | | |
| --- | --- | --- | --- | --- | --- | --- |
| **Gas flow rate** | **5 NLPM** | | **10 NLPM** | | **20 NLPM** | |
| **Pre** | 55,84 | 65,945 | 51,48 | 67,755 | 55,84 | 82,68 |
| **Post** | 64,425 | 107,7 | 67,4 | 102,55 | 74,075 | 118,7 |

Supplementary Table S3: Arterial carboxyhemoglobin (COHb) of the control (NBO) and the test (EHT) group during the recovery phase of the in vivo experiments. The values marked with an asterisk were not included in the calculation of the carboxyhemoglobin half-life of the EHT system, because the EHT system was stopped after 80 min.

|  | **Arterial COHb (%)** | | | | | |
| --- | --- | --- | --- | --- | --- | --- |
| **Time in recovery phase** | **NBO** | | | **EHT** | | |
| **0:00:00** | 35,4 | 42,5 | 41,1 | 42 | 42,3 | 39,8 |
| **0:15:00** | 30,2 | 35 | 31 | 31,1 | 29,3 | 29 |
| **0:30:00** | 26,2 | 31 | 25,4 | 24,9 | 21,7 | 21,5 |
| **0:45:00** | 23,3 | 27,3 | 21,6 | 20,9 | 12,9 | 13 |
| **1:00:00** | 20,4 | 24,6 | 14,7 | 15,3 | 9,4 | 9,5 |
| **1:15:00** | 15,1 | 22,5 | 12,5 | 13,5 | 7,5 | 6,9 |
| **1:30:00** | 12,9 | 20,2 | 10,6 | 12,4* | 5,7* | 5,4* |
| **1:45:00** | 11,4 | 15,3 | 9,2 | 10,8* | 4,3* | 4,1* |
| **2:00:00** | 10 | 13,8 | 7,8 | 10,2* | 3,5* | 3,2* |
| **2:15:00** | 9,3 | 12,3 | 6,7 | 8,8* | 2,5* | 2,7* |
| **2:30:00** | 8,1 | 11,2 | 5,9 | 7,5* | 2,5* | 1,8* |
| **2:45:00** | 7,3 | 10,2 | 5 | 6,7* | 1,7* | 1,3* |
| **3:00:00** | 6,4 | 9,2 | 4,3 | 6* | 1,6* | 1* |
| **3:15:00** | 5,6 | 8,4 | 3,9 | 5,5* | 1,3* | 0,5* |
| **3:30:00** | 5 | 7,7 | 3,2 | 4,8* | 0,9* | 0,4* |
| **3:45:00** | 4,6 | 6,9 | 2,7 | 4,3* | 0,8* | 0,1* |
| **4:00:00** | 4,1 | 6,5 | 2,5 | 3,9* | 0,3* | -0,1* |
| **4:15:00** | 3,5 | 5,7 | 2 | 3* | 0* | -0,3* |
| **4:30:00** | 3,1 | 5,4 | 1,6 | 2,8* | -0,1* | 3* |
| **4:45:00** | 2,9 | 5 | 1,5 | 2,4* | 0,1* | 2,9* |
| **5:00:00** | 2,6 | 4,4 | 1,1 | 2,5* | -0,3* | 2,7* |

Supplementary Table S4: Plasma free hemoglobin (PfHb) of the control (NBO) and the test (EHT) group at different times during the in vivo experiments: "Pre" at start of recovery phase, "Intra" at 60 minutes of recovery phase, "Post" directly after recovery phase, and "End" at termination of animal trial (360 minutes after start of recovery phase).

|  | **PfHb (mg/dL)** | | | | | | | |
| --- | --- | --- | --- | --- | --- | --- | --- | --- |
|  | **NBO** | | | | | **EHT** | | |
| **Pre** | | 18,45 | 14,885 | 10,026 | 15,365 | | 13,31 | 12,425 |
| **Intra** | | 12,515 | 15,96 | 10,97 | 30,98 | | 13,185 | 16,02 |
| **Post** | | 12,245 | 15,145 | 11,59 | 27,47 | | 12,63 | 14,27 |
| **End** | | 12,45 | 13,015 | 11,465 | 16,15 | | 9,444 | 9,669 |
